# Synthesis and Anticancer Activity Assessment of Zelkovamycin Analogues

**DOI:** 10.3390/molecules29184483

**Published:** 2024-09-21

**Authors:** Xinrong Xie, Hongshun Huang, Yogini S. Jaiswal, Shaoyang Su, Linxia Yang, Yu Fan, Yifu Guan, Leonard L. Williams, Hedong Bian

**Affiliations:** 1 Key Laboratory of Chemistry and Engineering of Forest Products (State Ethnic Affairs Commission), Guangxi Collaborative Innovation Center for Chemistry and Engineering of Forest Products, School of Chemistry and Chemical Engineering, Guangxi Minzu University, Nanning 530006, China; 2 Center for Excellence in Post Harvest Technologies, North Carolina Agricultural and Technical State University, The North Carolina Research Campus, Kannapolis, NC 28081, USA; 3 Department of Applied Chemistry Teaching and Research, Guangxi Vocational University of Agriculture, Nanning 530007, China

**Keywords:** cyclopeptide, zelkovamycins, analogue, antitumor activity

## Abstract

The zelkovamycin family is a class of cyclic octapeptides with potent antibacterial and antiviral activity. Due to their unique chemical structures and excellent bioactivity, zelkovamycins have consistently attracted the interest of synthetic chemists. However, only the total synthesis of zelkovamycin and zelkovamycin G has been reported until now. The current work presents, for the first time, the synthesis of zelkovamycin analogues, along with their anticancer activity assessment. Firstly, the corresponding chain peptide based on the amino acid sequence of zelkovamycin H was synthesized using the Fmoc solid-phase peptide strategy. This was followed by cyclization under high dilution conditions to obtain compound **21**, and its structure was elucidated by NMR analysis. The results confirm that compound **21** is not the natural product of zelkovamycin H. We deduced that during the synthesis of peptide **12**, the D-Abu residue epimerized to the L-Abu form, leading to the formation of peptide **20**, which blocked our efforts during the synthesis of zelkovamycin H. Two more analogues, **22** and **23,** were synthesized by changing the structure of amino acid residues using the same strategy. The anticancer activity of analogues **21**–**23** against Huh-7 cells was evaluated in vitro; however, their IC_50_ values were >50 μM.

## 1. Introduction

**Figure 1:**
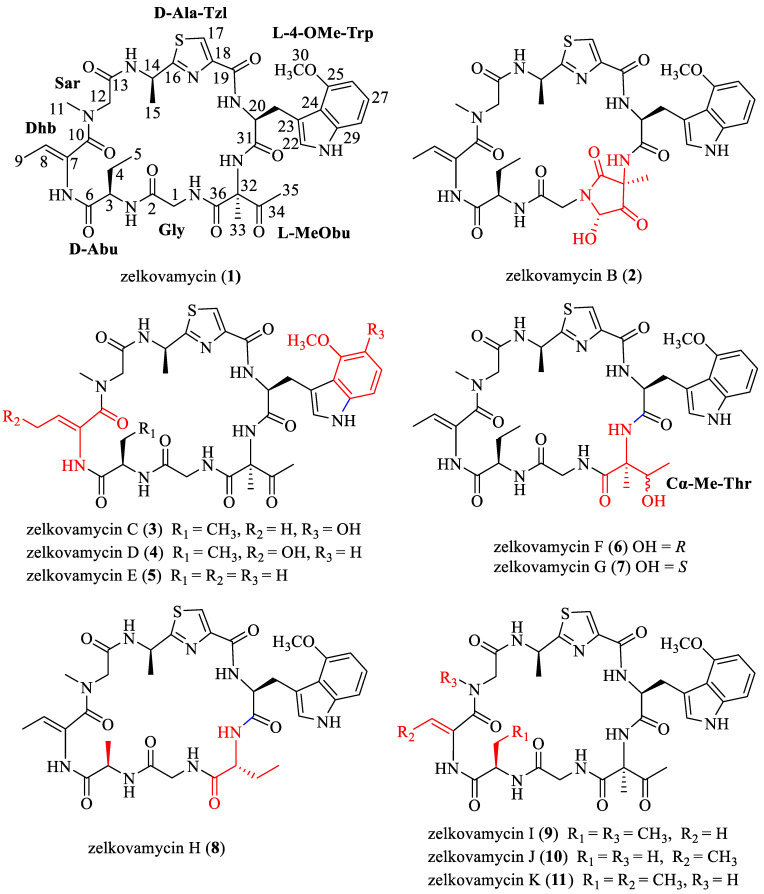
Structure of zelkovamycins.

Natural products are an exceptional source of inspiration in drug discovery and frequently serve as valuable starting points in chemical probe synthesis [1,2]. Zelkovamycin (**1**, Figure 1) is a cyclic octapeptide consisting of glycine (Gly), 2-amino butyric acid (Abu), (Z)-dehydrobutyric acid (Z-Dhb), sarcosine (Sar), alanine–thiazole amino acid (Ala-Thz), 4-methoxy tryptophan (4-MeO-Trp), and 2-methyl-3-oxobutyrine (MeObu) amino acids (for unprecedented amino acids in a peptide natural product). It was discovered by the Ōmura research group in 1999 from *Streptomyces* sp. K96-0670 [3,4]. The stereochemistry of the different amino acids in zelkovamycin, however, was not reported in the original report. In 2021, Chan et al. found *Actinomadura graeca* sp. as a novel producer of zelkovamycin [5]. Gan et al. identified 10 other zelkovamycin congeners from the culture of endophytic *Kitasatospora* sp. CPCC 204717, named zelkovamycins B–K (**2**–**11**, Figure 1) [6,7]. From these cyclopeptides, zelkovamycin B was featured by an unprecedented 3-methyl-5-hydroxypyrrolidine-2,4-dione ring system linked to the cyclopeptide skeleton. Zelkovamycins F and G (**6** and **7**) possess unprecedented nonproteinogenic amino acid residues, namely L-α-methylthreonine and L-α-methyl-*allo*-threonine, respectively. These represent the first example of natural peptide products that contain the Cα-methyl-threonine monomer. Compared to the structure of other zelkovamycins, zelkovamycin H (**8**) was the only congener lacking the oxy group at C-34 and the α-methyl group at C-32. The incorporation of the Abu residue by replacing the MeObu unit in zelkovamycin H might be due to the low selectivity of the A domain in module 8 for biosynthetic gene cluster of zelkovamycins from *Kitasatospora* sp. CPCC 204717 [6].

In the literature, zelkovamycins are reported to have antibacterial and antiviral activity [6,7]. Compounds **1** and **4** were the most potent antibiotics against *S. epidermidis*, while **1** displayed the most potent activity against *Staphylococcus aureus* and *S. aureus* compared to other zelkovamycins. Compounds **6**, **7** and **9** showed potent antibacterial activity against methicillin-resistant *Staphylococcus aureus* and *Staphylococcus epidermidis*. Compound **5** exhibited potent inhibitory activity against the H1N1 virus [EC_50_ 0.3 μM, selective index (SI) = 149], 150- and 50-fold stronger than compound **1** and ribavirin (positive control), respectively. Compounds **1** and **5** exhibited significant antiviral activity against the hepatitis C virus. Recently, compound **1** was found to be a potent oxidative phosphorylation (OXPHOS) inhibitor, showing potential in cancer therapy [8]. Zelkovamycin is similar to the argyrin family, both consisting of 4-methoxytryptophan, an alanine–thiazole moiety, sarcosine, glycine, and α-aminobutyric acid [9,10,11]. Argyrins possess diverse biological activities, including immunosuppressive, antibacterial, and cytotoxic effects, and so on [9,10,11,12,13,14,15,16]. Given its structural similarity to the argyrin family, zelkovamycins are likely to exhibit comparable biological activity [17].

Due to their unique structure and promising biological properties, zelkovamycins have attracted considerable attention from chemists. The Kaiser research group achieved the first total synthesis of zelkovamycin in 2020 [8]. Its previously proposed molecular structure was revised, and the stereochemistry of Abu, Ala-Thz, and 4-MeO-Trp residues was confirmed by total synthesis. Firstly, zelkovamycin G (**7**) was synthesized by a [2 + 2 + 3] fragment splicing strategy, and then the complete synthesis of zelkovamycin was achieved with a Dess–Martin oxidation reaction.

So far, the chemical synthesis of other zelkovamycins has not been reported. Due to the unusual features of zelkovamycins and the lack of knowledge of their bioactivities, there is a need for their total synthesis study. Herein we report the synthesis of zelkovamycin analogues for the first time and the evaluation of their anticancer activity.

## 2. Results and Discussion

### 2.1. Synthesis of Zelkovamycin H

**Figure 2:**
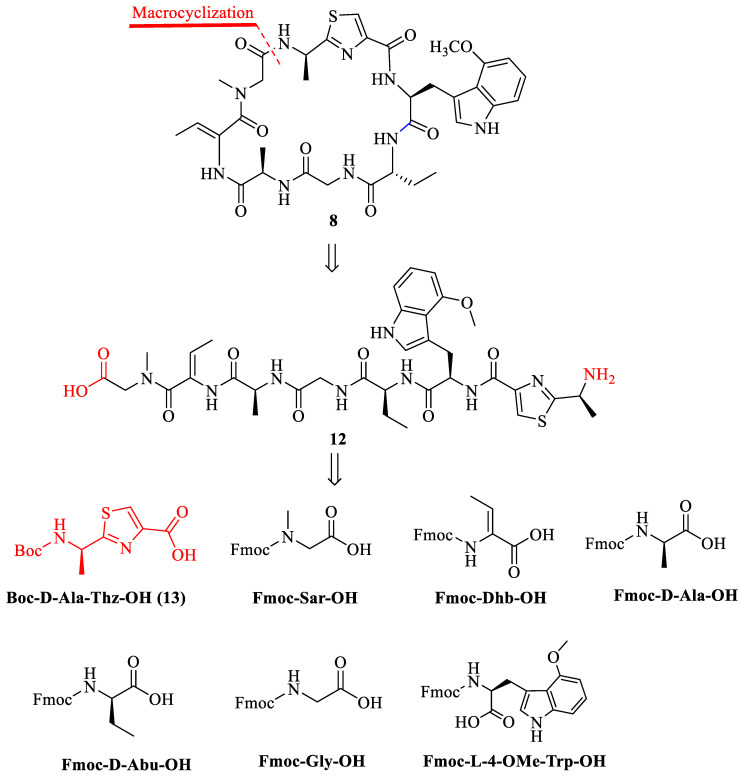
Retrosynthetic analysis of zelkovamycin H.

The retrosynthetic analysis of zelkovamycin H (**8**) is shown in Figure 2. Heptapeptide **12** can be obtained by cleaving the amide bond between Sar and Ala-Tzl residues. This heptapeptide can be assembled by the following amino acids: Fmoc-Sar-OH, Fmoc-Dhb-OH, Fmoc-D-Ala-OH, Fmoc-Gly-OH, Fmoc-D-Abu-OH, Fmoc-L-4-OMe-Trp-OH, and Boc-D-Ala-Thz-OH. All these amino acids are commercially available, except for Boc-D-Ala-Thz-OH, which is a thiazole-containing amino acid.

**Scheme 1:**
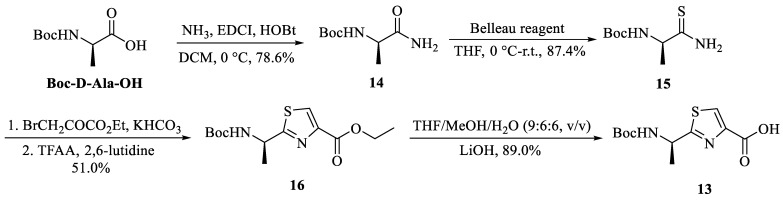
Synthetic route of Boc-D-Ala-Thz-OH **13**.

To synthesize heptapeptide **12**, the first step was to obtain amino acid **13**. For the synthesis of building block **13**, a straightforward route from Boc-D-Ala-OH that involved transformation to thioamide **15** via the intermediate amide and thiolation with Belleau’s reagent (Scheme 1) was followed [18,19]. Compound **15** was then reacted with ethyl bromopyruvate to obtain an intermediate, that on treatment with trifluoroacetic anhydride and 2,6-lutidine, gave the thiazole **16**. This reaction was carried out via a modified Hantzsch thiazole synthesis leading to a 51.0% yield [20]. It was then hydrolyzed to obtain the key amino acid **13** with an 89.0% yield [21].

After obtaining amino acid **13**, the heptapeptide **12** was assembled. Solid phase peptide synthesis (SPPS) is preferred due to its simplicity in assembling peptide chains, rapid reaction times, and straightforward post-processing with repeated washes. These attributes make the process less time-consuming, economic, and enable one to employ the SPPS method for heptapeptide **12**. Two strategies for the SPPS exist, the Boc/benzyl and Fmoc/*t*Bu strategy. In the Boc/benzyl strategy, the α-amino group is protected by the Boc group and the side chain functional groups by benzyl-based protecting groups. The Boc group is removed by trifluoroacetic acid (TFA), whereas the side chain protecting groups require hydrogen fluoride (HF) for deprotection [22]. The α-amino group is protected by the base–labile Fmoc group and side chain functional groups by the acid–labile *t*Bu or trityl-based protecting groups in the Fmoc/*t*Bu strategy [23]. The Fmoc group is removed by piperidine, and the side chain protecting groups are cleaved using TFA. Since the Boc/benzyl strategy requires the use of HF for final deprotection, the Fmoc/*t*Bu strategy is the most widely adopted. The 2-chlorotrityl chloride (2-CTC), which most often comes attached to a polystyrene resin, is well suited for the synthesis of peptide acids [24]. Moreover, protected peptide acids can be cleaved from the resin using 1% TFA or 10–20% acetic acid.

**Scheme 2:**
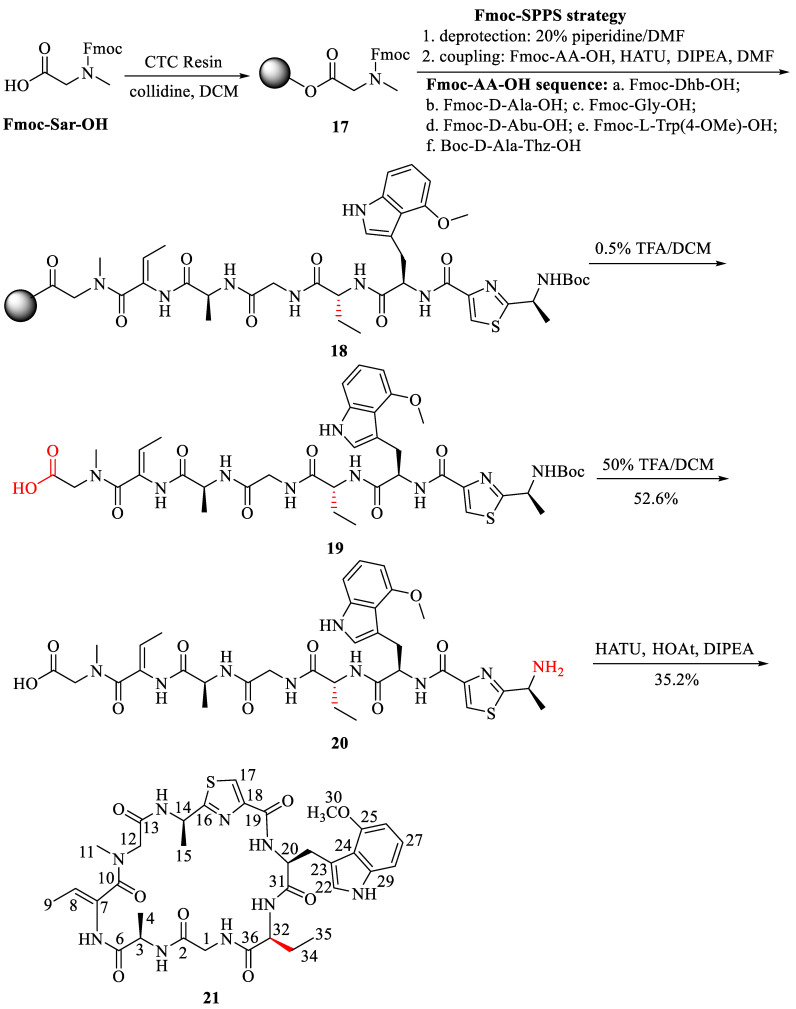
Synthetic route of compound **21**.

Therefore, the synthesis of heptapeptide **12**, following the Fmoc/*t*Bu strategy, began with the attachment of Fmoc-Sar-OH to the 2-CTC resin via its carboxyl group, using collidine as the base. This led to the first amino acid-loaded resin **17** (Scheme 2) [25]. Solid-phase peptide elongation was performed using the standard Fmoc/*t*Bu protocol: removal of the Fmoc group with 20% piperidine/DMF, followed by acylation of the resulting amine with Fmoc amino acid using HATU/DIPEA as the coupling reagent [26]. Incomplete coupling was observed when DCC/HOBt, HBTU, or EDCI/HOBt were used as coupling reagents. Solid-phase peptide elongation, following the deprotection and coupling cycle according to the amino acid sequence shown in Scheme 2, provided resin-supported heptapeptide **18**. Finally, cleavage of the heptapeptide **18** from the resin and removal of the Boc protecting group with trifluoroacetic acid (TFA) produced heptapeptide **20**. It was then cyclized without further purification to give cyclopeptide **21**, with HATU/HOAt/DIPEA used as the condensation reagent, in a highly diluted solution. The overall yield of **21** from the initial Fmoc-Sar-OH was 6.5%.

The ^13^C NMR chemical shifts of zelkovamycin H and compound **21** are presented in Table 1. While most chemical shifts are consistent, there are significant discrepancies in Δδ for C-4, C-12, C-30, and C-32, with the largest Δδ of 2.6 observed for C-12. This indicates that compound **21** is not the natural product of zelkovamycin H. The ^13^C NMR chemical shifts for chiral centres C-3, C-14, and C-20 are generally consistent. However, the Δδ in chiral C-32 is 1, suggesting that the configuration of this chiral centre in D-Abu residue from the Fmoc-D-Abu-OH has been epimerized to L-Abu form in the coupling reaction (HATU/DIPEA). As a result, the desired peptide **12** was not obtained; instead, peptide **20** was produced during the SPPS. The racemization of the D-Abu residue may be attributed to the steric hindrance of the adjacent L-4-OMe-Trp residue.

### 2.2. Synthesis of Analogue

**Figure 3:**
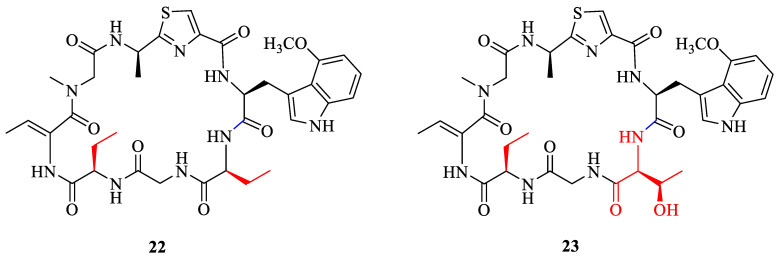
Structures of analogues.

Compound **1** demonstrated the most potent activity against *Staphylococcus aureus* and *Staphylococcus epidermidis* with MICs of 3.1 and 1.6 μM, respectively. In contrast, Compound **5**, which only differs from **1** by the substitution of Ala instead of Abu, exhibited a 4- to 32-fold reduction in activity against these strains. Compounds **6** and **7** displayed potent antibacterial activities against methicillin-resistant *S. aureus* (MRSA) ATCC33591 and *S. epidermidis*, with MICs ranging from 0.62 to 2.5 μM. However, Compound **8**, which incorporates the D-Abu residue instead of MeObu, showed no activity (MIC > 160 μg/mL) against 7 strains. To obtain more structure–activity relationship information, two other analogues **22**–**23** were prepared to investigate the importance of the amino acid residue (Figure 3). Based on the structure of **8**, analogue **22** replaces Ala residues with Abu residues, because this change reflects the zelkovamycin structure. Analogue **23** substitutes the Cα-Me-Thr-OH residue of **6** with the Thr-OH residue. These analogues share the same basic skeleton structure and were synthesized using the same method, with Fmoc-Sar-OH as the first amino acid and 2-CTC resin as the carrier. The overall yields of **22** and **23** from the initial Fmoc-Sar-OH were 4.8% and 5.6%, respectively.

### 2.3. Anticancer Evaluation

The cytotoxicity of compounds **21**–**23** was evaluated by MTT assay against Huh-7 cancer cells (Table 2). Our synthetic compounds **21**–**23** exhibited low cytotoxicity (IC_50_ > 50 μM), whereas the positive control 5-fluorouracil had an intermediate-level cytotoxic (IC_50_ = 15.3 μM) effect. The results showed that these synthetic peptides had poor anticancer activity.

## 3. Materials and Methods

### 3.1. General Chemical Procedures

^1^H and ^13^C NMR spectra were recorded on a Bruker Avance-III 400 MHz spectrometer (Billerica, MA, USA), in the indicated solvent. Chemical shifts (*δ*) were expressed in ppm with reference to the solvent signals (CDCl_3_; ^1^H: 7.26 ppm; ^13^C: 77.00 ppm), and coupling constants (*J*) were reported in Hz. All NMR experiments were obtained using standard pulse sequences supplied by the vendor. Column chromatography (CC) was carried out on silica gel (200–400 mesh, Qingdao Marine Chemical Factory, Qingdao, China). Thin-layer chromatography was performed on Whatman glass-backed plates coated with 0.25 mm layers of silica gel 60. Reversed-phase flash chromatography was performed on an Agilent 1260 Infinity (Santa Clara, CA, USA) with ODS-A semi-preparative column (YMC-Pack, 5 μm, 250 mm × 10 mm). ESI-MS was recorded on a Micromass QTOF-2 (Milford, MA, USA). HepG2 cell lines were obtained from Shanghai Cell Bank (Shanghai, China). All commercially available reagents were purchased from commercial suppliers and used without further purification. All reagents or solvents were reagent or HPLC grade.

### 3.2. Synthesis of Boc-D-Ala-Thz-OH (**13**) [18,19,20,21]

Compound **14**: Boc-D-Ala-OH (3.0 g, 15.9 mmol) and HOBt (2.2 g, 15.9 mmol) were dissolved in dichloromethane (DCM, 90.0 mL). At 0 °C, EDCI (3.8 g, 20.0 mmol) was added, and the mixture was stirred at room temperature for 2 h. After cooling the mixture back to 0 °C, a solution of ammonia in methanol (79.5 mL, 2 mol/L in MeOH) was added dropwise. The reaction mixture was allowed to warm to room temperature and stirred for 1 h. The reaction was then extracted with DCM and washed sequentially with H_2_O (10.0 mL) and brine (10.0 mL). The organic layer was dried with Na_2_SO_4_ and concentrated in vacuo. The resulting residue was purified by silica gel column chromatography (V_DCM_:V_MeOH_ = 25:1) to afford amide **5** (white solid, 2.4 g, 78.6% yield). ^1^H NMR (400 MHz, CDCl_3_) δ (ppm): δ 6.27 (s, 1H), 5.67 (s, 1H), 5.08 (s, 1H), 4.20 (s, 1H), 1.43 (s, 9H), 1.36 (d, *J* = 7.20 Hz, 3H).

Compound **15**: To a stirred solution of compound **14** (2.0 g, 10.6 mmol) in tetrahydrofuran (THF, 85.0 mL), Belleau reagent (3.4 g, 6.3 mmol) was added at 0 °C. The temperature was then gradually increased to room temperature, and the mixture was stirred for 2 h. The reaction mixture was poured into ice (80.0 g) and saturated NaHCO_3_ solution (80.0 mL). It was extracted with ethyl acetate (EtOAc, 3 × 100.0 mL), washed with brine (40.0 mL), and dried over anhydrous sodium sulphate. After concentrating, the residue was purified using column chromatography (V_DCM_:V_MeOH_ = 5:1), yielding a white solid (1.9 g, 87.4% yield). ^1^H NMR (400 MHz, DMSO-d_3_) δ (ppm): 9.56 (s, 1H), 9.08 (s, 1H), 6.81 (d, *J* = 7.31 Hz, 1H), 4.25 (m, 1H), 1.37 (s, 9H), 1.24 (d, *J* = 7.23 Hz, 3H).

Compound **16**: Ethyl bromopyruvate (3.3 mL) was added to a suspension of compound **15** (1.8 g, 8.8 mmol) and potassium bicarbonate (KHCO_3_, 7.1 g, 71.0 mmol), which was stirred in dimethoxyethane (DME, 15.0 mL) at −15 °C for 5 min. After 1 min, the reaction mixture was treated with a solution of trifluoroacetic anhydride (TFAA, 5.0 mL) and 2,6-lutidine (8.8 mL) in DME (4.5 mL) at −15 °C, and stirred for 4 h at the same temperature. The reaction was quenched with water (70.0 mL) and extracted with EtOAc (3 × 100.0 mL). The combined organic layer extract was washed with brine (40.0 mL) and dried with anhydrous sodium sulphate (Na_2_SO_4_). The mixture was concentrated and purified using column chromatography (V_DCM_:V_MeOH_ = 8:1), yielding a white solid (1.4 g, 51.0% yield). ^1^H NMR (400 MHz, CDCl_3_) δ (ppm): 8.09 (s, 1H), 5.27 (s, 1H), 5.11 (s, 1H), 4.41 (dd, *J* = 14.4, 7.32 Hz, 2H), 1.62 (d, *J* = 6.92 Hz, 3H), 1.45 (s, 9H), 1.40 (t, *J* = 7.92 Hz, 3H).

Compound **13**: Compound **16** was dissolved in methanol (3.0 mL), THF (4.5 mL), and H_2_O (3.0 mL) at 0 °C. Lithium hydroxide (LiOH, 0.5 N, 18.0 mL) was added and the mixture was stirred for 6 h. The pH was adjusted to 2, using saturated KHSO_4_. The mixture was extracted with EtOAc (3 × 100.0 mL), washed with brine (40.0 mL), dried over anhydrous sodium sulphate, and concentrated in vacuo. Chromatographic separation of the residue (V_DCM_:V_MeOH_ = 10:1) on the silica gel column of the extract afforded compound **13** (white solid, 1.1 g, 89.0% yield). ^1^H NMR (400 MHz, CDCl_3_) δ (ppm): 8.21 (s, 1H), 5.30 (s, 1H), 5.12 (s, 1H), 1.64 (d, *J* = 7.25 Hz, 3H), 1.43 (s, 9H).

### 3.3. Synthesis of Analogue **21** {Cyclo-[(Sar-Dhb-Ala-Gly-Abu-Trp(4-OMe)-Ala(Thz)]}

#### 3.3.1. Loading First Amino Acid Fmoc-Sar-OH to 2-CTC Resin for the Synthesis of **17**

2-CTC resin (0.3 g) was pre-soaked with DCM for 1 h, and then DCM was filtered. A solution of Fmoc-Sar-OH (0.3 g, 1.1 mmol) and collidine (0.3 mL, 2.3 mmol) in DCM (8.0 mL) was added to the resin. The mixture was shaken at room temperature for 8 h. Later, the reaction solution was filtered, and the resin was washed successively with DCM (5.0 mL × 3) and DMF (5.0 mL × 3). A solution of DCM (8.5 mL), MeOH (1.0 mL), and DIPEA (0.5 mL) was added to the resin for capping the free amino group of resin and then washed alternately with DCM (5.0 mL × 3) and DMF (5.0 mL × 3) to give **17**.

#### 3.3.2. Synthesis of Chain Peptide on the Resin (**18**)

Deprotection of Fmoc group: A 20% piperidine/DMF solution (5.0 mL) was added to **17** and shaken for 15 min at room temperature. After this step, the resin was washed again alternately with DCM (5.0 mL × 3) and DMF (5.0 mL × 3).

Coupling reaction: Next, a mixture of Fmoc-Dhb-OH (0.3 g, 1.1 mmol), HATU (0.4 g, 1.1 mmol), and DIPEA (0.6 mL, 3.4 mmol) in DMF (2.0 mL) was added to the resin of deprotected Fmoc group and shaken for 3 h. The reaction mixture was filtered, and the resin was washed thrice alternately with DCM (5.0 mL × 3) and DMF (5.0 mL × 3).

The deprotection of the Fmoc group and coupling steps were repeated, sequentially coupling the remaining amino acids to the resin in the following order: Fmoc-D-Ala-OH, Fmoc-Gly-OH, Fmoc-D-Abu-OH, Fmoc-L-Trp(4-OMe)-OH, and Boc-D-Ala-Thz-OH. Then, **18** was obtained.

#### 3.3.3. Cleavage of Peptide from Resin to Synthesize **19**

**18** was initially treated with TFA/DCM (0.5% 7.5 mL). After shaking for 20 min, the TFA/DCM solution was filtered and collected. The resin was then washed with DCM (20.0 mL × 3), and the DCM washes were collected. This procedure was repeated thrice. The combined organic solutions were concentrated under reduced pressure to yield a pale-yellow solid **19**, which was used directly for the next step without purification.

#### 3.3.4. Macrolactamization to Synthesize **21**

The N-terminal protecting group (Boc) of **19** (86 mg, 0.1 mmol) was dissolved in a DCM-TFA (DCM/TFA = 1/1, 8.0 mL) mixture in an ice bath, and the mixture was stirred for 1 h at this temperature, and then DCM and TFA were removed to give **20** (40 mg, 52.6%), which was then used directly for the next step, without purification. The peptide **20** (0.05 mmol, 45.0 mg) was dissolved in DCM (100.0 mL) and stirred vigorously in an ice bath. Condensation agents HATU (0.1 mmol, 45.0 mg) and HOAt (0.1 mmol, 16.0 mg) were dissolved in DMF (1.0 mL) and added dropwise to the reaction mixture. DIPEA (4.5 eq, 0.25 mmol, 50.0 μL) was added at 0 °C, and the reaction was allowed to proceed at room temperature for 48 h. The reaction was quenched with 0.1 M HCl aq. (50.0 mL). The crude reaction mixture was extracted with DCM (3 × 60.0 mL), followed by saturated sodium bicarbonate solution (2 × 20.0 mL), and brine (2 × 30.0 mL). The organic layers were combined, dried over anhydrous Na_2_SO_4_, filtered, and concentrated in vacuo. The crude material was purified by preparative HPLC [flow rate: 2 mL/min; elution: C_2_H_5_CN/H_2_O = 75/25 (0.0–60.0 min)] to afford compound **21** (13.0 mg, 34.2%).

**21**: ^1^H NMR (400 MHz, CDCl_3_) δ 9.48 (s, 1H), 8.90 (d, *J* = 8.5 Hz, 1H), 8.50 (d, *J* = 7.4 Hz, 1H), 8.05 (d, *J* = 1.5 Hz, 1H), 7.15 (d, *J* = 6.7 Hz, 1H), 7.05 (d, *J* = 7.9 Hz, 1H), 7.01 (d, *J* = 2.9 Hz, 1H), 6.91 (d, *J* = 7.7 Hz, 1H), 6.84 (d, *J* = 8.1 Hz, 1H), 6.53 (d, *J* = 7.8 Hz, 1H), 5.50–5.47 (m, 1H), 5.40 (d, *J* = 6.7 Hz, 1H), 5.20 (d, *J* = 7.4 Hz, 1H), 4.98 (d, *J* = 16.9 Hz, 1H), 4.09 (d, *J* = 3.7 Hz, 1H), 4.05 (d, *J* = 3.9 Hz, 1H), 4.00 (s, 3H), 3.74 (d, *J* = 8.0 Hz, 1H), 3.53 (s, 1H), 3.41 (s, 1H), 3.13 (d, *J* = 12.9 Hz, 3H), 1.90–1.85 (m, 3H), 1.76 (d, *J* = 7.3 Hz, 1H), 1.72 (d, *J* = 7.2 Hz, 3H), 1.43 (d, *J* = 6.9 Hz, 3H), 0.95–0.90 (m, 3H).

^13^C NMR (10 MHz, CDCl_3_) δ 171.7, 171.3, 171.2, 169.8, 169.4, 169.0, 167.1, 160.0, 153.4, 150.2, 136.6, 130.4, 124.8, 122.8, 122.6, 116.3, 111.0, 106.4, 105.8, 99.8, 57.7, 55.5, 52.5, 51.1, 48.5, 45.5, 41.8, 37.6, 28.2, 21.4, 20.7, 14.1, 11.4, 11.3. ESIMS: *m*/*z* 760.3 [M + Na]^+^ (calcd for C_34_H_43_N_9_NaO_8_S, 760.3)

### 3.4. Synthesis of the Analogues

The synthetic procedure for analogues **22**–**23** was carried out following the same method (Section 3.3.1, Section 3.3.2, Section 3.3.3 and Section 3.3.4) as used for compound **21**. For analogue **22** {cyclo-[(Sar-Dhb-Abu-Gly-Abu-Trp(4-OMe)-Ala(Thz)]}, the starting amino acid was Fmoc-Sar-OH, and the amino acids were sequentially coupled in the following order: Fmoc-Dhb-OH, Fmoc-D-Abu-OH, Fmoc-Gly-OH, Fmoc-D-Abu-OH, Fmoc-L-Trp(4-OMe)-OH and Boc-D-Ala-Thz-OH. The crude material was purified by preparative HPLC [flow rate: 2 mL/min; elution: C_2_H_5_CN/H_2_O = 75/25 (0.0–50.0 min)].

For analogue **23** {cyclo-[(Sar-Dhb-Abu-Gly-Thr-Trp(4-OMe)-Ala(Thz)]}, the synthesis also began with Fmoc-Sar-OH as the starting amino acid. The amino acids were then connected in the following order: Fmoc-Dhb-OH, Fmoc-D-Abu-OH, Fmoc-Gly-OH, Fmoc-D-Thr-OH, Fmoc-L-Trp(4-OMe)-OH and Boc-D-Ala-Thz-OH. The crude material was purified by preparative HPLC [flow rate: 2 mL/min; elution: C_2_H_5_CN/H_2_O = 75/25 (0.0–30.0 min)].

**22**: ^1^H NMR (400 MHz, CDCl_3_, *J* in Hz) δ (ppm): 10.73 (s, 1H), 9.38 (s, 1H), 8.91 (d, *J* = 8.52 Hz, 1H), 8.59 (d, *J* = 7.40 Hz, 1H), 8.03 (s, 1H), 7.57–7.38 (m, 1H), 7.11–6.95 (m, 3H), 6.83 (d, *J* = 8.12 Hz, 1H), 6.52 (d, *J* = 7.78 Hz, 1H), 5.59–5.43 (m, 2H), 5.28–5.13 (m, 2H), 5.00 (d, *J* = 16.93 Hz, 1H), 4.17 (d, *J* = 15.30 Hz, 1H), 4.12–4.02 (m, 1H), 3.99 (s, 3H), 3.75 (dd, *J* = 17.48, 7.92 Hz, 1H), 3.58 (dd, *J* = 15.50, 3.40 Hz, 1H), 3.51–3.37 (m, 2H), 3.10 (s, 3H), 2.27–2.09 (m, 1H), 2.07–1.95 (m, 1H), 1.89 (d, *J* = 7.08 Hz, 3H), 1.72 (d, *J* = 7.09 Hz, 3H), 1.63 (s, 3H), 0.95–0.86 (m, 6H);

^13^C NMR (100 MHz, CDCl_3_) δ171.9, 171.7, 171.4, 169.5, 169.5, 169.2, 167.3, 160.2, 153.6, 150.5, 136.9, 130.7, 125.1, 122.8, 122.7, 116.5, 110.7, 106.5, 106.0, 100.1, 57.9, 55.7, 54.6, 52.7, 51.2, 45.6, 42.0, 37.6, 28.5, 21.6, 21.3, 21.0, 11.5, 11. 5, 10.5. HRESITOFMS: *m*/*z* 774.3012 [M + Na]^+^ (calcd for C_35_H_45_N_9_NaO_8_S, 774.3004).

**23**: ^1^H NMR (400 MHz, CDCl_3_, *J* in Hz) δ (ppm): 10.73 (s, 1H), 9.38 (s, 1H), 8.91 (d, *J* = 8.52 Hz, 1H), 8.59 (d, *J* = 7.40 Hz, 1H), 8.03 (s, 1H), 7.60–7.55 (m, 1H), 7.11–6.95 (m, 3H), 6.83 (d, *J* = 8.12 Hz, 1H), 6.52 (d, *J* = 7.78 Hz, 1H), 5.59–5.43 (m, 2H), 5.28–5.13 (m, 2H), 5.00 (d, *J* = 16.93 Hz, 1H), 4.17 (d, *J* = 15.30 Hz, 1H), 4.12–4.02 (m, 1H), 3.99 (s, 3H), 3.75 (dd, *J* = 17.48, 7.92 Hz, 1H), 3.58 (dd, *J* = 15.50, 3.40 Hz, 1H), 3.51–3.37 (m, 2H), 3.10 (s, 3H), 2.27–2.09 (m, 1H), 2.07–1.95 (m, 1H), 1.89 (d, *J* = 7.08 Hz, 3H), 1.72 (d, *J* = 7.09 Hz, 3H), 1.63 (s, 3H), 0.95–0.86 (m, 6H);

^13^C NMR (100 MHz, CDCl_3_) δ 173.1, 171.5, 171.4, 169.8, 169.3, 169.2, 167.3, 160.5, 153.8, 150.0, 136.7, 130.6, 124.9, 123.2, 122.9, 116.3, 110.9, 106.6, 105.7, 99.7, 77.5, 77.2, 76.8, 66.9, 62.3, 55.5, 54.7, 52.9, 51.3, 45.5, 41.6, 37.7, 28.3, 21.3, 21.2, 20.7, 11.5, 10.7. HRESITOFMS: *m*/*z* 790.2959 [M + H]^+^ (calcd for C_35_H_45_N_9_NaO_9_S, 790.2953).

### 3.5. Anticancer Assays

Huh-7 cells were cultured in 1% penicillin-streptomycin mixture DMEM and (DMEM) medium containing 10% fetal bovine serum (FBS) at 37 °C, under 5% CO_2_ atmosphere. The medium was refreshed every 2–3 days, and cells in the near-confluency phase [27] at densities of 5000 cells/100.0 μL/well fresh culture medium, were typically passaged 3 to 4 times, before use in experiments.

The cells were detached with 0.25% trypsin and the suspension was inoculated overnight into 96-well culture plates. The target compound was dissolved in DMSO at a concentration of 20.0 μM, and DMSO was used as a blank control. About 10.0 μL of the sample and control solutions were sampled in 5 wells each and incubated for 24 h. The medium was discarded and replenished with sterile PBS. To each well 10.0 μL of DMEM complete medium was added, followed by 10.0 μL of MTT (5.0 mg/mL) solution and incubation at 37 °C under 5% CO_2_ atmosphere for 4 h. About 150.0 μL DMSO was added to each well, to lyse the Methazan precipitate. The optical density values (OD) were determined using a microplate reader at 570 nm with a reference wavelength of 450 nm. The corresponding concentration of cell proliferation inhibition rate was calculated according to the inhibition rate formula. All experiments were performed in triplicate to obtain the final values.

## 4. Conclusions

In summary, an efficient solid-phase synthesis of zelkovamycin analogues was achieved for the first time. The process began with the preparation of the crucial building block Boc-D-Ala-Thz-OH **13** through ammonolysis, vulcanization, cyclization, and hydrolysis using Boc-Ala-OMe as the starting material. Subsequently, peptide **20** was synthesized by SPPS, using 2-chlorotriphenylmethyl chloride resin as the carrier. Compound **21** was then obtained through macrocyclization using a liquid-phase method. However, NMR analysis shows that compound **21** is not the natural zelkovamycin H. Additionally, two more analogues, **22** and **23**, were synthesized by the same method. Anticancer activity was evaluated, revealing that the synthesized analogues exhibited poor anticancer activity. Changing amino acid residues did not significantly influence their anticancer activity.

## Figures and Tables

**Table 1:** Comparison of ^13^C NMR spectroscopic data obtained for natural zelkovamycin H and compound **21** in CDCl_3_.

No.	Amino Acid Residue	Natural (150 MHz)	Synthetic (100 MHz)	Δδ
1	Gly	42.0	41.8	−0.2
2		171.6	171.3	−0.3
3	Ala	51.3	51.1	−0.2
4		15.4	14.1	1.3
6		169.6	169.8	0.2
7	Dhb	130.6	130.4	−0.2
8		110.5	110.0	−0.5
9		11.4	11.4	0
10		169.0	169.0	0
11	Sar	37.4	37.6	0.2
12		51.1	48.5	−2.6
13		167.1	167.1	0
14	Ala-Tzl	45.4	45.5	0.1
15		20.9	20.7	−0.2
16		171.2	171.2	0
17		122.8	122.8	0
18		150.3	150.2	−0.1
19		160.0	160.0	0
20	4-Meo-Trp	52.6	52.5	−0.1
21		28.1	28.2	0.1
22		125.0	124.8	−0.2
23		106.3	106.4	0.1
24		116.3	116.3	0
25		153.3	153.4	0.1
26		100.0	99.8	−0.2
27		122.5	122.6	0.1
28		105.9	105.8	−0.1
29		136.7	136.6	−0.1
30		55.6	57.7	2.1
31		171.7	171.7	0
32	Abu	54.5	55.5	1.0
34		21.1	21.4	0.3
35		10.4	11.3	0.9
36		169.4	169.4	0

**Table 2:** Cytotoxic activity of analogues in Huh-7 cells ^a^.

Compound	IC_50_ (24 h)
**21**	56.2
**22**	62.8
**23**	75.7
5-fluorouracil	15.3

^a^ Results are expressed as IC_50_ values in micromolar and data were obtained from three individual experiments.

## Data Availability

Data are contained within the article and Supplementary Materials.

## Supplementary Materials

The following supporting information can be downloaded at https://www.mdpi.com/article/10.3390/molecules29184483/s1. Figure S1. ^1^H NMR of compound **14**; Figure S2. ^1^H NMR of compound **15**; Figure S3. ^1^H NMR of compound **16**; Figure S4. ^1^H NMR of compound **13**; Figure S5. ^1^H NMR of compound **21**; Figure S6. ^13^C NMR of compound **21**; Figure S7. ^1^H NMR of compound **21**; Figure S8. ^13^C NMR of compound **22**; Figure S9. ^1^H NMR of compound **23**; Figure S10. ^13^C NMR of compound **23**; Figure S11. ESIMS of compound of compound **21**; Figure S12. HRESITOFMS of compound **22**; Figure S13. HRESITOFMS of compound **23**; Figure S14. Preparative HPLC of compound **21**; Figure S15. Preparative HPLC of compound **22**; Figure S16. Preparative HPLC of compound **23**.

## References

[1] Harvey A.L., Edrada-Ebel R., Quinn R.J. (2015). The Re-Emergence of Natural Products for Drug Discovery in the Genomics Era. Nat. Rev. Drug Discov..

[2] Song X., Ku C.F., Si T.X., Jaiswal Y.S., Williams L.L., Lu D.Y., Huang J.J., He Z.D., Wang M.Z. (2022). Synthesis and Biological Activities Assessment of 4-, 6-, and 9-Phenylphenalenone Derivatives. ChemistrySelect.

[3] Zhang H., Tomoda H., Tabata N., Oohori M., Shinose M., Takahashi Y., Omura S. (1999). Zelkovamycin, a New Cyclic Peptide Antibiotic from *Streptomyces* sp. K96-0670. I. Production, Isolation and Biological Properties. J. Antibiot..

[4] Tabata N., Tomoda H., Zhang H., Uchida R., Omura S. (1999). Zelkovamycin, a New Cyclic Peptide Antibiotic from *Streptomyces* sp. K96-0670 II. Structure Elucidation. J. Antibiot..

[5] Tarantini F.S., Brunati M., Taravella A., Carrano L., Parenti F., Hong K.W., Williams P., Chan K.G., Heeb S., Chan W.C. (2021). *Actinomadura graeca* sp. nov.: A novel producer of the macrocyclic antibiotic zelkovamycin. PLoS ONE.

[6] Hao X.M., Yu J.Q., Wang Y.J., Connolly J.A., Liu Y.F., Zhang Y.Q., Yu L.Y., Cen S., Goss R.J.M., Gan M.L. (2020). Zelkovamycins B-E, Cyclic Octapeptides Containing Rare Amino Acid Residues from an Endophytic *Kitasatospora* sp. Org. Lett..

[7] Hao X.M., Li S.S., Wang G.Y., Li J.R., Peng Z.G., Zhang Y.Q., Yu L.Y., Gan M.L. (2022). Zelkovamycins F and G, Cyclopeptides with Cα-Methyl-Threonine Residues, from an Endophytic *Kitasatospora* sp. J. Nat. Prod..

[8] Krahn D., Heilmann G., Vogel F.C.E., Papadopoulos C., Zweerink S., Kaschani F., Meyer H., Roesch A., Kaiser M. (2020). Zelkovamycin is an OXPHOS Inhibitory Member of the Argyrin Natural Product Family. Chem. Eur. J..

[9] Sasse F., Steinmetz H., Schupp T., Petersen F., Memmert K., Hofmann H., Heusser C., Brinkmann V., Matt P.V., Höfle G. (2002). Argyrins, Immunosuppressive Cyclic Peptides from Myxobacteria I. Production, isolation, physico-chemical and biological properties. J. Antibiot..

[10] Vollbrecht L., Steinmetz H., Höfle G. (2002). Argyrins, Immunosuppressive Cyclic Peptides from Myxobacteria II. Structure Elucidation and Stereochemistry. J. Antibiot..

[11] Pogorevc D., Tang Y., Hoffmann M., Zipf G., Bernauer H.S., Popoff A., Steinmetz H., Wenzel S.C. (2019). Biosynthesis and Heterologous Production of Argyrins. ACS Synth. Biol..

[12] Nickeleit I., Zender S., Sasse F., Geffers R., Brandes G., Sörensen I., Steinmetz H., Kubicka S., Carlomagno T., Menche D. (2008). Argyrin A Reveals a Critical Role for the Tumor Suppressor Protein p27^kip1^ in Mediating Antitumor Activities in Response to Ptoteasome Inhibition. Cancer Cell.

[13] Stauch B., Simon B., Basile T., Schneider G., Malek N.P., Kalesse M., Priv-Doz T.C. (2010). Elucidation of the Structure and Intermolecular Interaction of a Reversible Cyclic-Peptide Inhibitor of the Proteasome by NMR Spectroscopy and Molecular Modeling. Angew. Chem. Int. Ed..

[14] Allardyce D.J., Bell C.M., Loizidou E.Z. (2019). Argyrin B, a non-competitive inhibitor of the human immunoproteasome exhibiting preference for β1i. Chem. Biol. Drug Des..

[15] Nyfeler B., Hoepfner D., Palestrant D., Kiiby C.A., Whitehead L., Yu R., Deng D., Cauhglan R.E., Woods A.L., Jones A.K. (2012). Identification of Elongation Factor G as the Conserved Cellular Target of Argyrin B. PLoS ONE.

[16] Bieleck P., Hüsecken P.L.K., Dötsch A., Steinmetz H., Hartmann R.W., Müller R., Häussler S. (2012). Mutation in Elongation Factor G Confers Resistance to the Antibiotic Argyrin in the Opportunistic Pathogen *Pseudomonas aeruginosa*. ChemBioChem.

[17] Romano G., Almeida M., Coelho A.V., Cutignano A., Gonçalves L.G., Hansen E., Khnykin D., Mass T., Ramsak A., Rocha M.S. (2022). Biomaterials and Bioactive Natural Products from Marine Invertebrates: From Basic Research to Innovative Applications. Mar. Drugs.

[18] Kigoshi H., Yamada S. (1999). Synthesis of Dolastatin I, a Cytotoxic Cyclic Hexapeptide from the Sea Hare Dolabella Auricularia. Tetrahedron.

[19] Lajoie G., Lepine F., Maziak L., Belleau B. (1983). Facile Regioselective Formation of Thiopeptide Linkages from Oligopeptides with New Thionation Reagents. Tetrahedron Lett..

[20] Aguilar E., Meyers A.I. (1994). Reinvestigation of a Modified Hantzsch Thiazole Synthesis. Tetrahedron Lett..

[21] Erik S., Robert-Franz-Xaver K., Nediljko B., Markus K. (2018). Painting argyrins blue: Negishi cross-coupling for synthesis of deep-blue tryptophan analogue β-(1-azulenyl)-L alanine and its incorporation into argyrin C. Bioorgan. Med. Chem..

[22] Merrifield R.B. (1963). Solid phase peptide synthesis I. The synthesis of a tetrapeptide. J. Am. Chem. Soc..

[23] Atherthon E., Fox H., Harkiss D., Logan C.J., Sheppard R.C., Williams B.J. (1978). A mild procedure for solid phase peptide synthesis: Use of fluorenylmethyloxycarbonylamino-acids. J. Chem. Soc. Chem. Commun..

[24] Barlos K., Chatzi O., Gatos D., Stavropoulos G. (1991). 2-chlorotrityl chloride resin. Studies on anchoring of Fmoc-amino acids and peptide cleavage. Int. J. Pep. Protein Res..

[25] Lopez J., Beck J., Bucher C., Berthelmann A., Markos S., Eissler S. (2022). Missing Link: Enabling Loading of 2-Chloride Resin in N-Butylpyrrolidinone as a Green Solvent. Org. Process Res. Dev..

[26] Carpino L.A., EI-Faham A. (1994). Effect of Tertiary Bases on O-Benzotriazolyluronium Salt-Induced Peptide Segment Coupling. J. Org. Chem..

[27] Qiu S., Gussem E.D., Tehrani K.A., Sergeyev S., Bultinck P., Herrebout W. (2013). Stereochemistry of the Tadalafil Diastereoisomers: A Critical Assessment of Vibrational Circular Dichroism, Electronic Circular Dichroism, and Optical Rotatory Dispersion. J. Med. Chem..

