# Supplementary material for: Synthesis and Anticancer Activity Assessment of Zelkovamycin Analogues
Source: Molecules. 2024 Sep 21;29(18):4483. doi: 10.3390/molecules29184483 (PMC11433887; doi:10.3390/molecules29184483)
Supplement: Supplementary file 1 [file molecules-29-04483-s001.zip › molecules-3203008-supplementary.pdf]

Synthesis and anticancer activity assessment of zelkovamycins analogues

Xinrong Xie [1,†], Hongshun Huang [1,†], Yogini S. Jaiswal [2,†], Shaoyang Su [3], Linxia Yang [1,†], Yu Fan [1], Yifu Guan [1,*], Leonard L. Williams [2,*], Hedong Bian [1,*]

[1]Key Laboratory of Chemistry and Engineering of Forest Products (State Ethnic Affairs Commission), Guangxi Collaborative Innovation Center for Chemistry an Engineering of Forest Products, School of Chemistry and Chemical Engineering, Guangxi Minzu University, Nanning, Guangxi 530006, China

[2]Center for Excellence in Post Harvest Technologies, North Carolina Agricultural and Technical State University, The North Carolina Research Campus, Kannapolis, NC 28081, USA

[3]Department of Applied Chemistry Teaching and Research, Guangxi Vocational University of Agriculture, Nanning 530007, China

*Corresponding author.

E-mail addresses: guanyifu@gxmzu.edu.cn (Y. G.), llw@ncat.edu (L. W.), bianhd@gxmzu.edu.cn (H. B.).

[†]These authors contributed equally to this work.

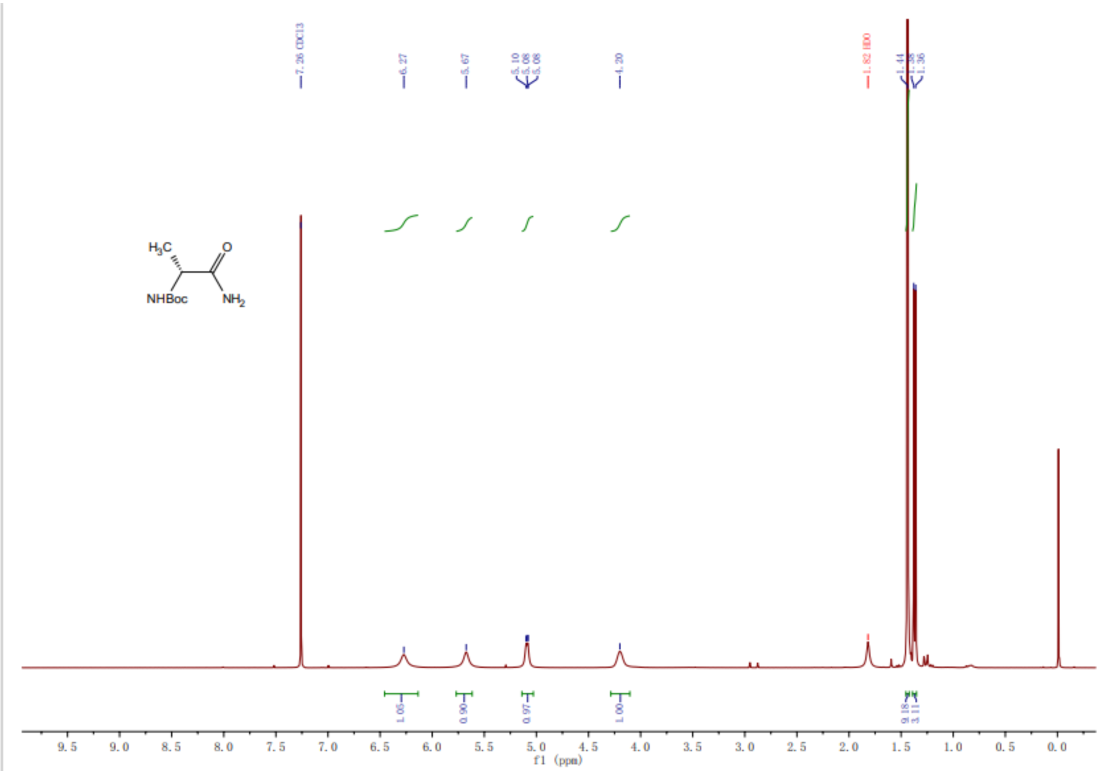

Figure S1 ^{1}H NMR of compound **14**

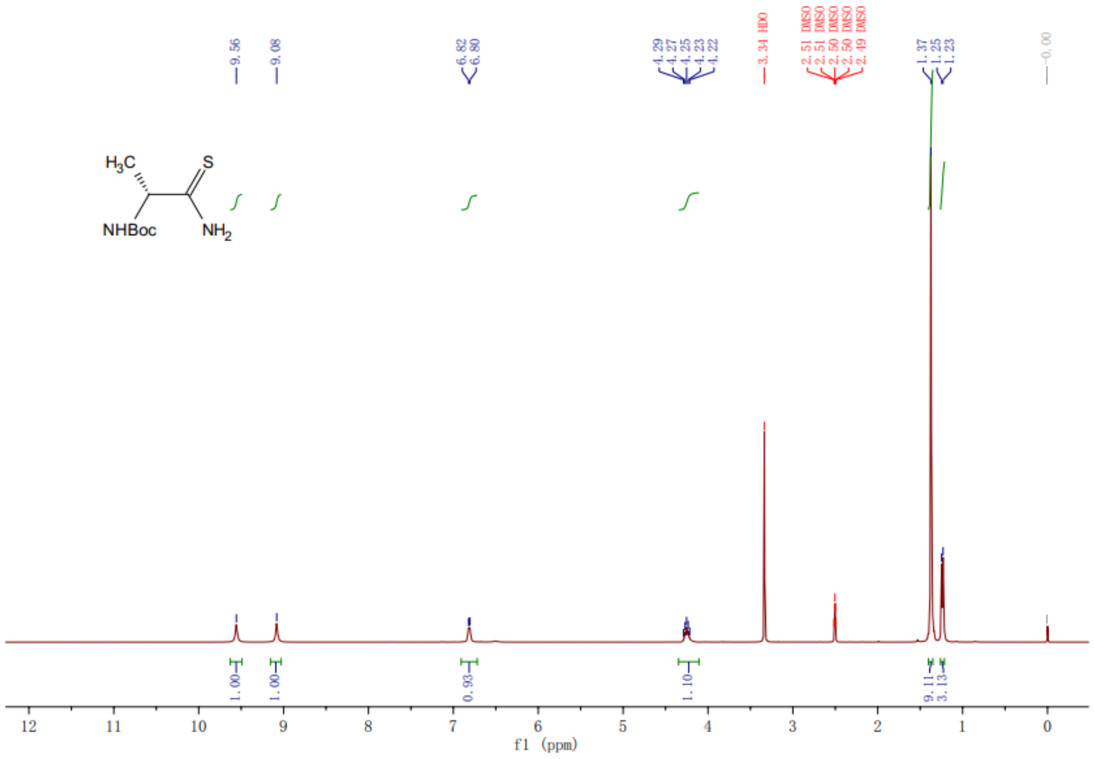

Figure S2 ^{1}H NMR of compound **15**

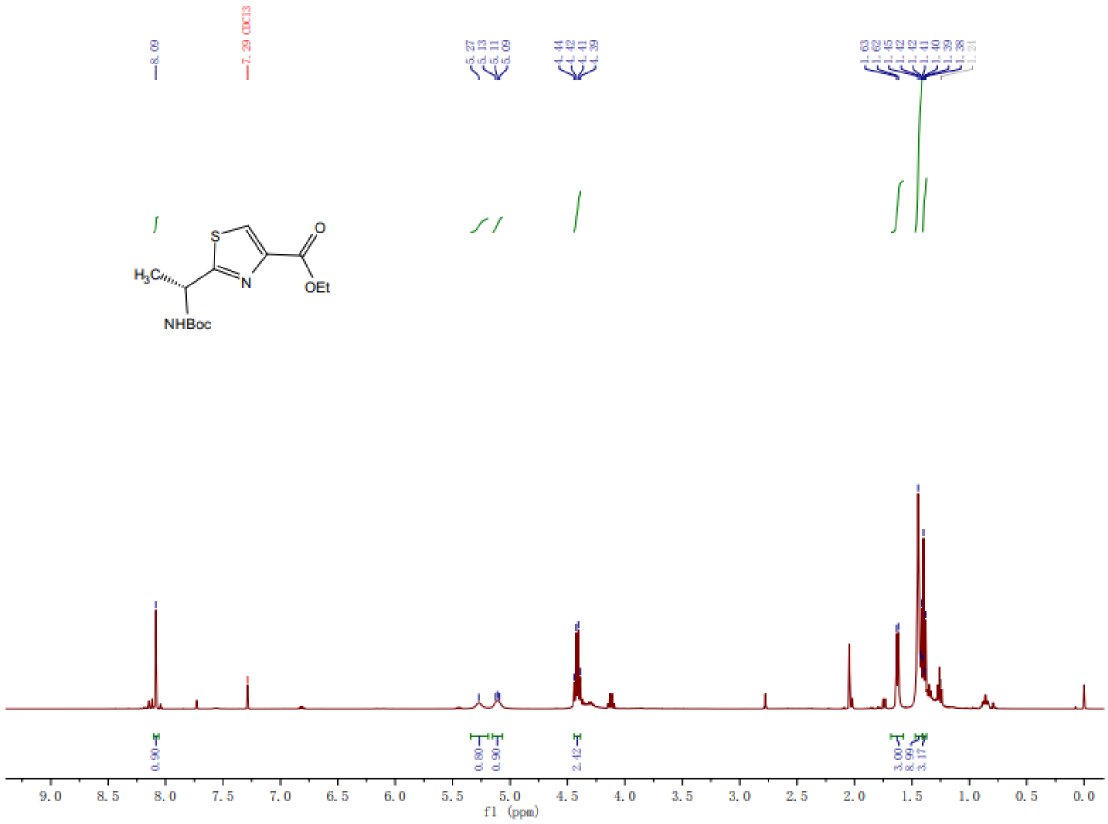

Figure S3 ^{1}H NMR of compound **16**

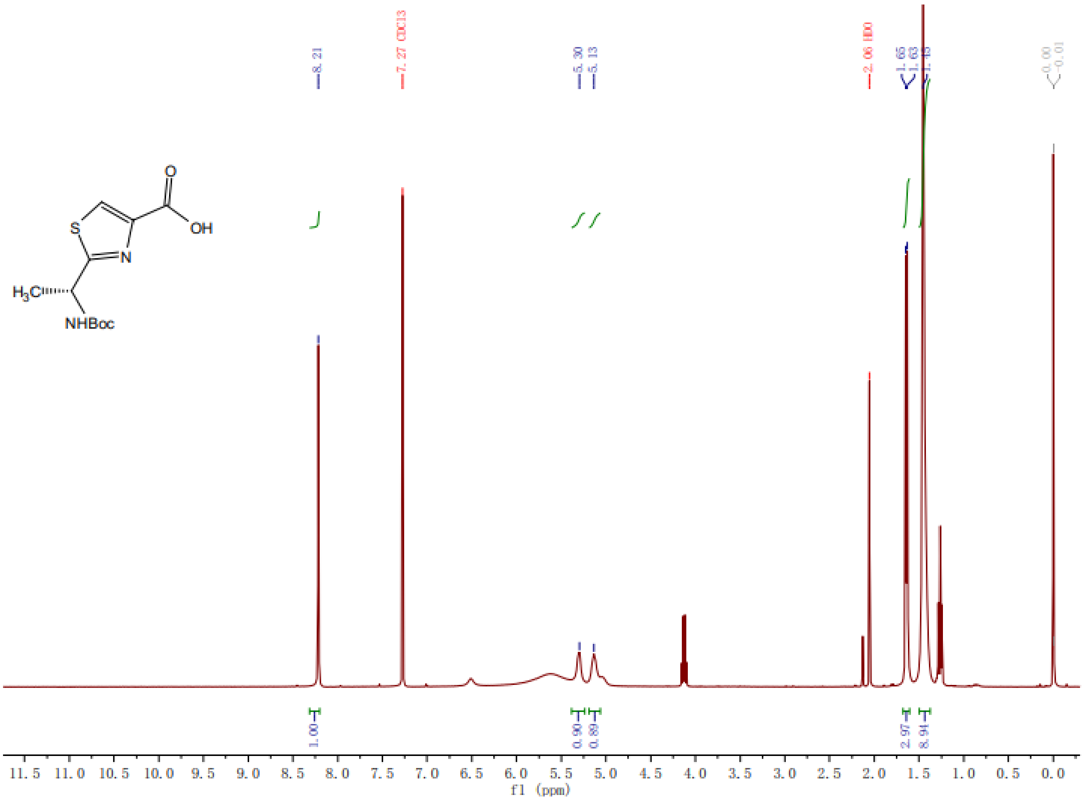

Figure S4 ^{1}H NMR of compound **13**

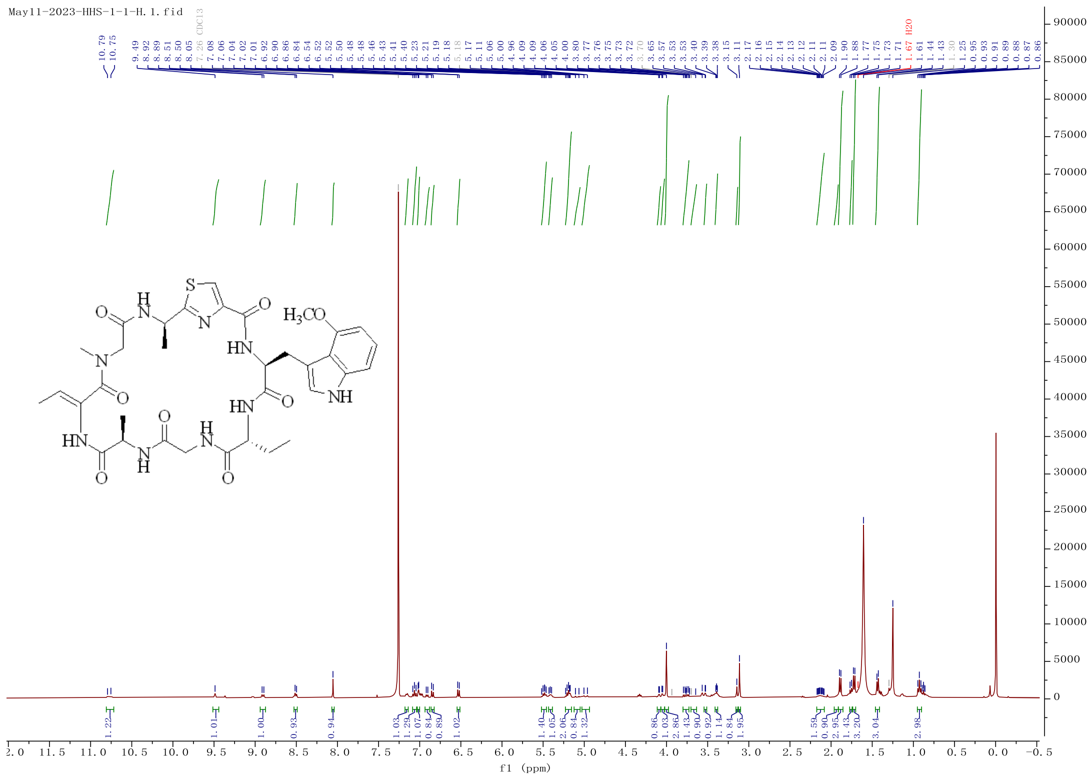

Figure S5 ^{1}H NMR of compound **21**

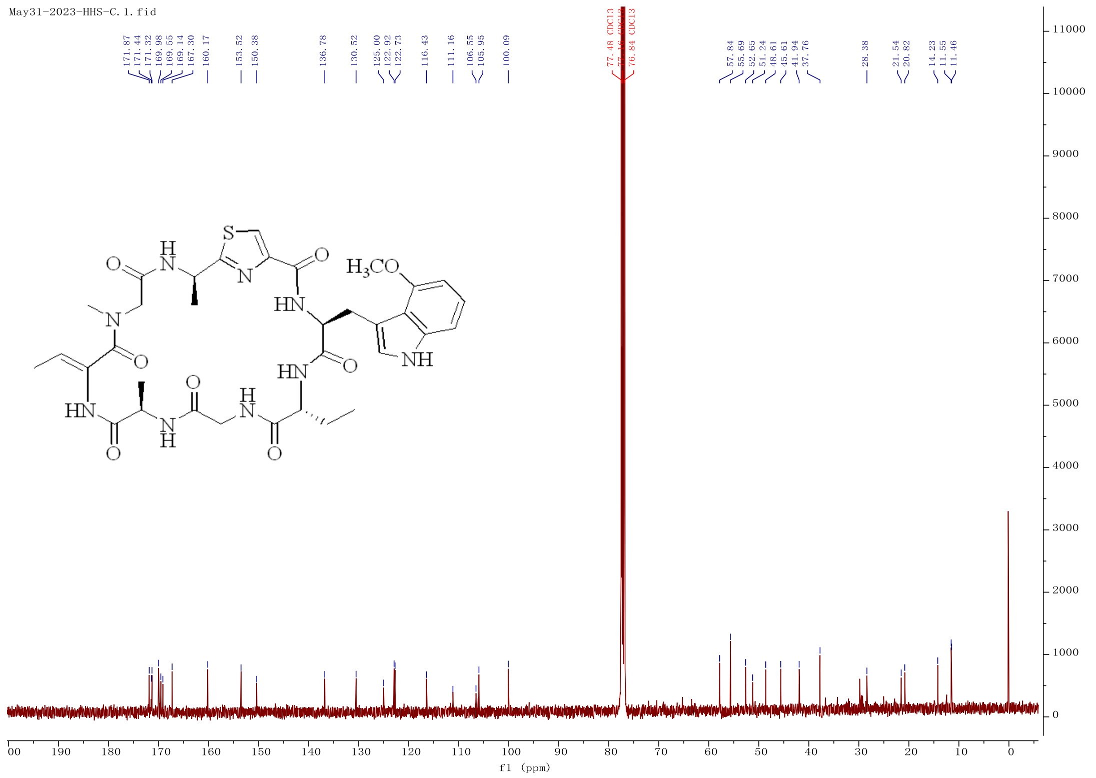

Figure S6 ^{13}C NMR of compound **21**

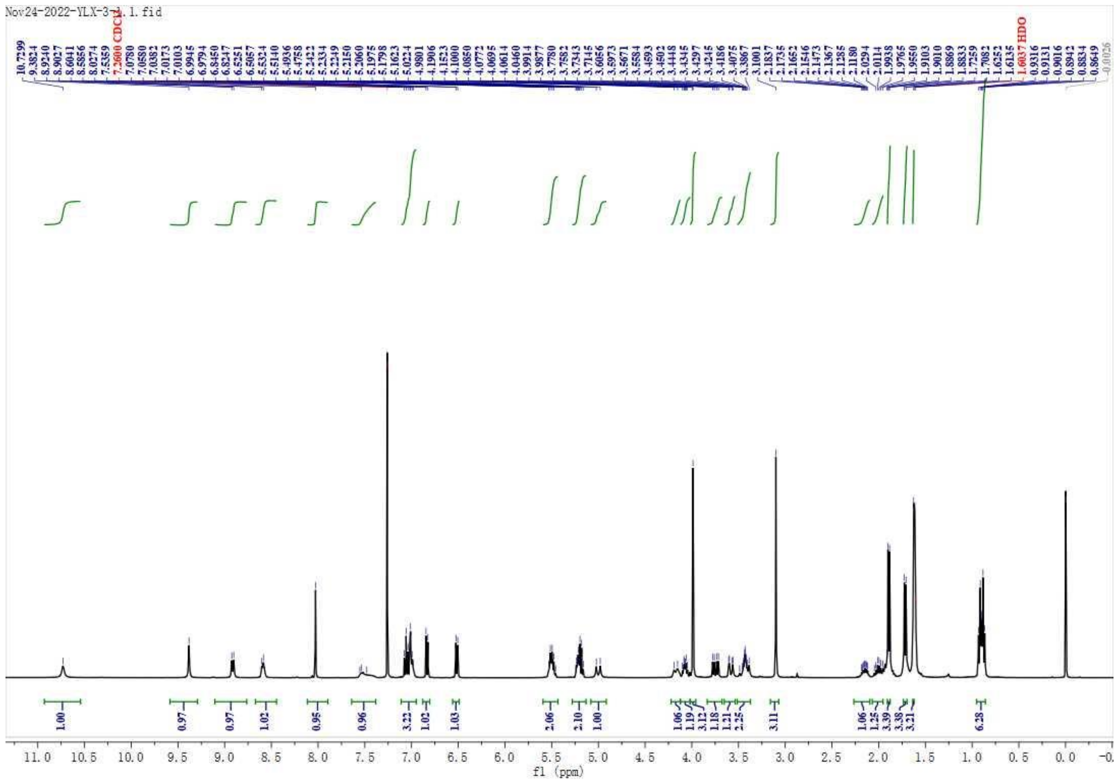

Figure S7 ^{1}H NMR of compound **22**

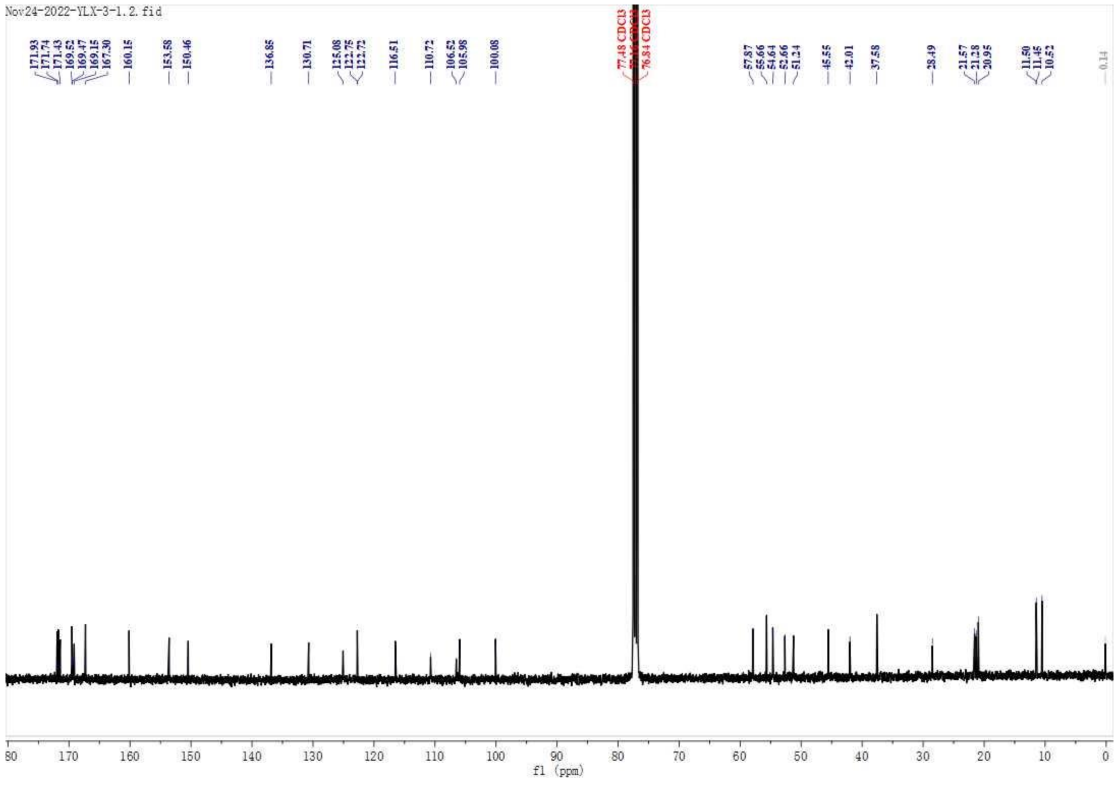

Figure S8 ^{13}C NMR of compound **22**

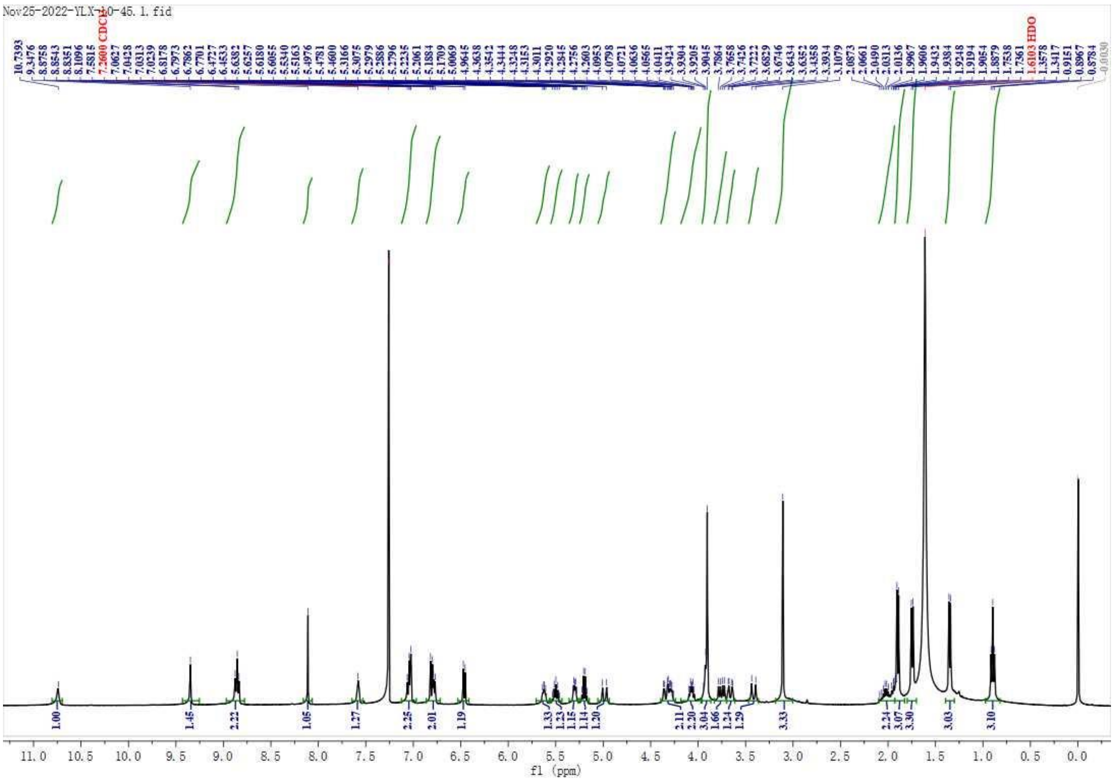

Figure S9 ^{1}H NMR of compound **23**

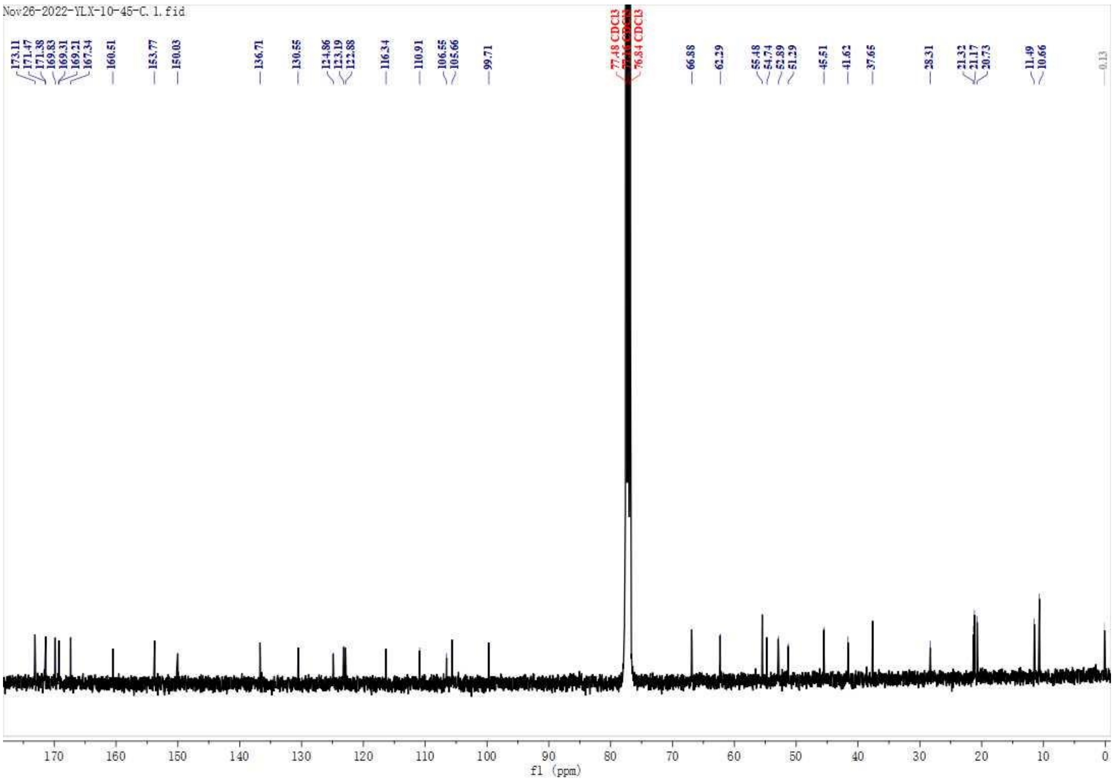

Figure S10 ^{13}C NMR of compound **23**

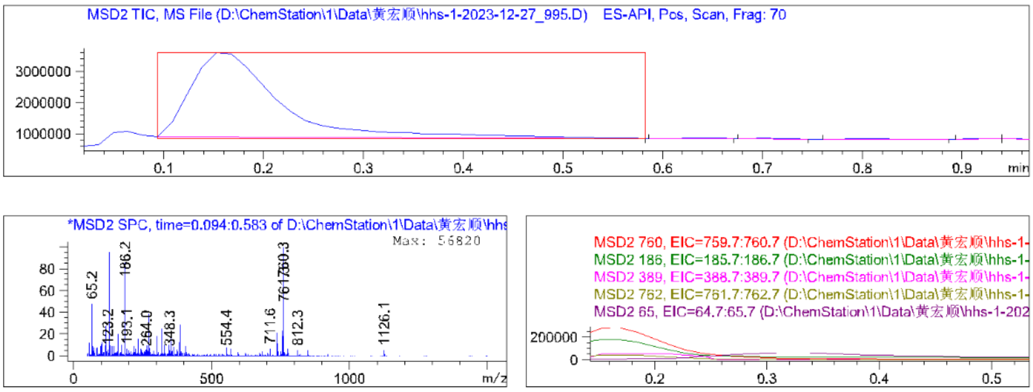

Figure S11 ESIMS of compound of compound **21**

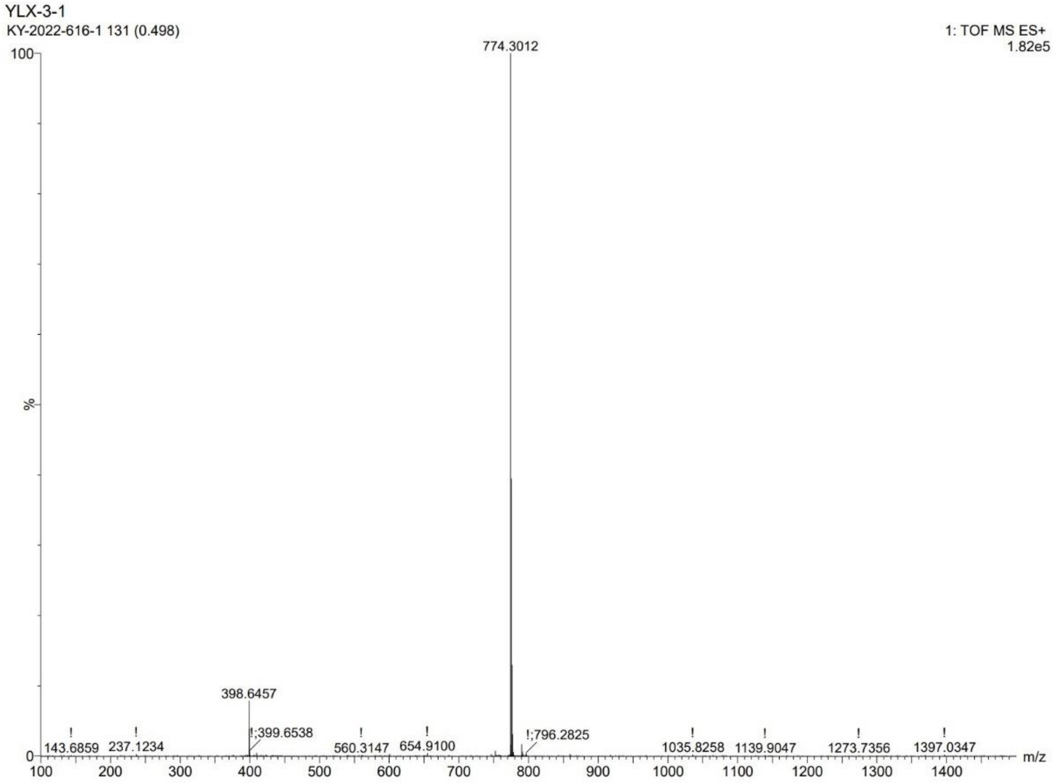

Figure S12 HRESITOFMS of compound **22**

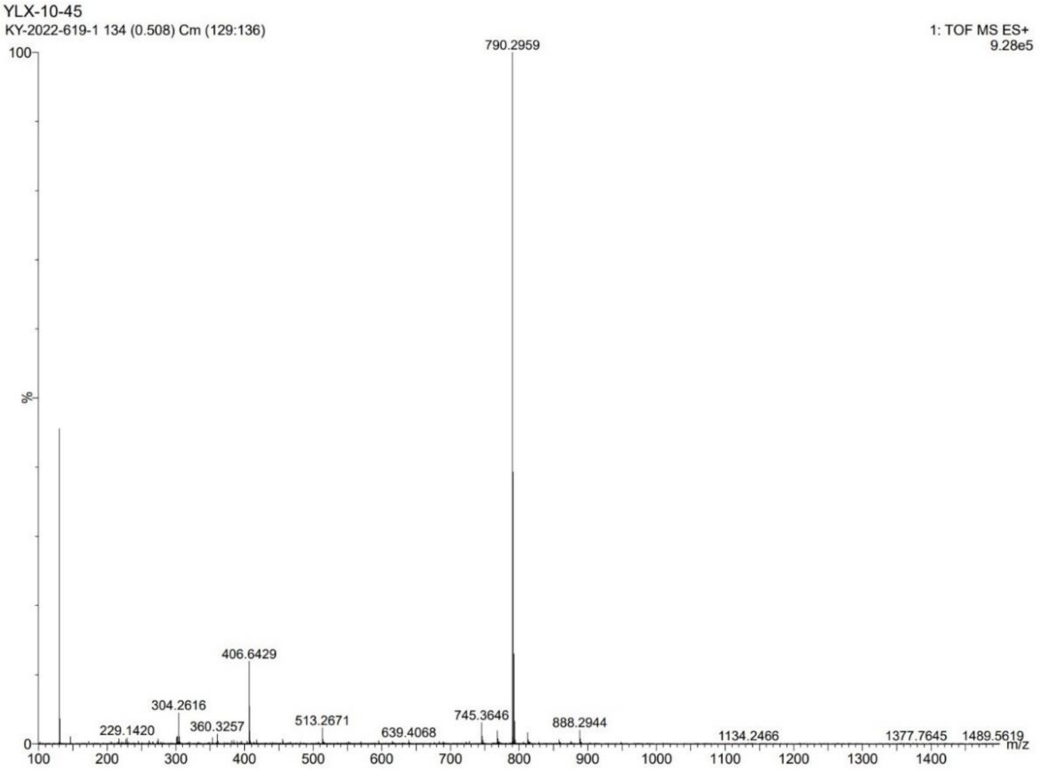

Figure S13 HRESITOFMS of compound **23**

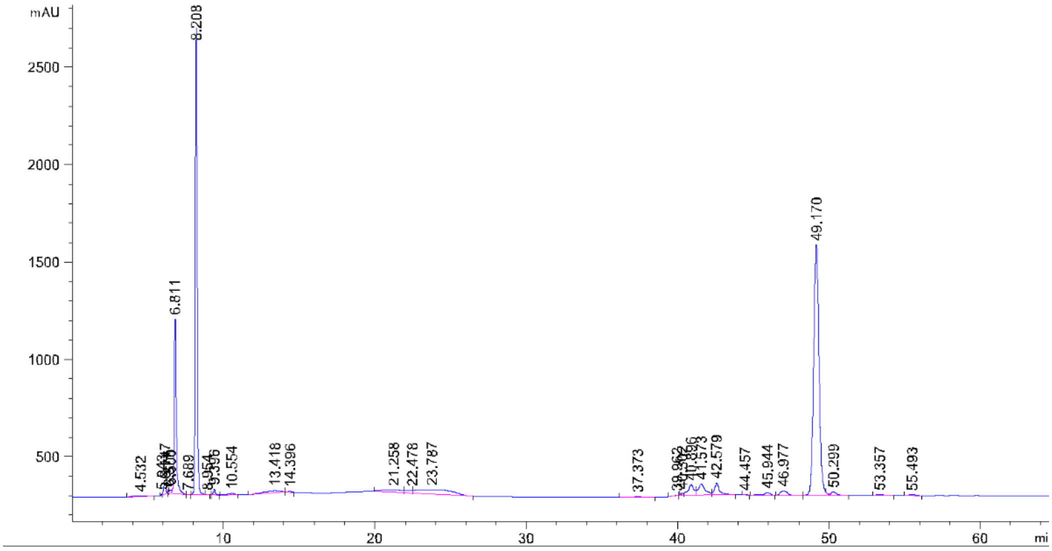

Figure S14 preparative HPLC of compound **21** (product: 49.17 min)

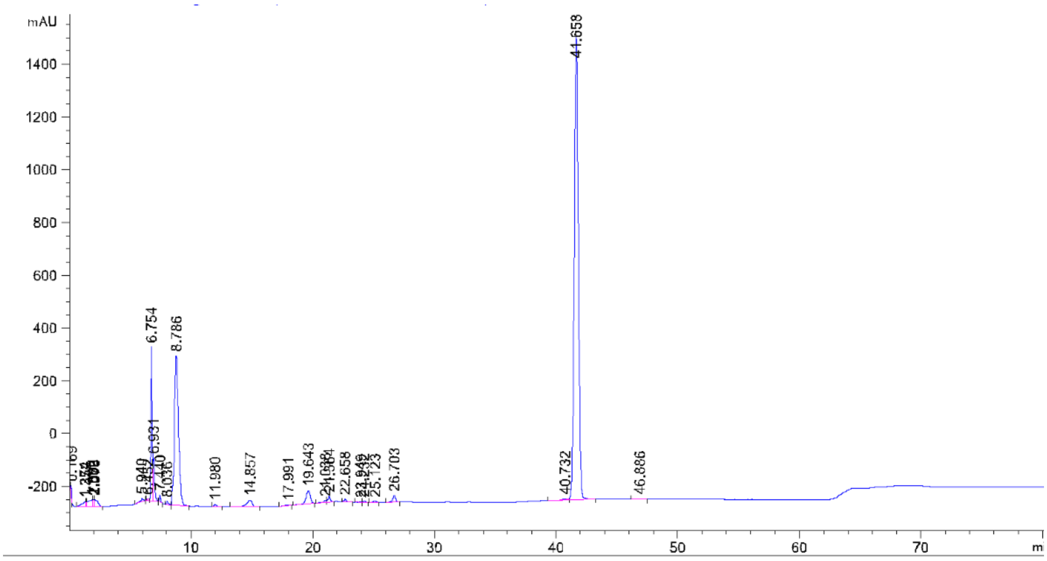

Figure S15 preparative HPLC of compound **22** (product: 41.66 min)

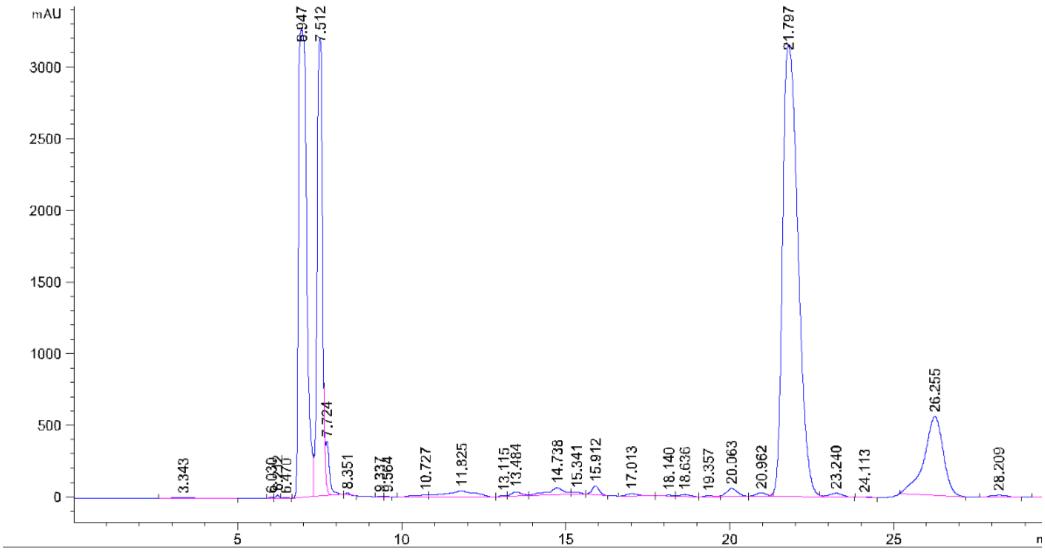

Figure S16 preparative HPLC of compound **23** (product: 21.80 min)
